# 4-Aminoquinoline: a comprehensive review of synthetic strategies

**DOI:** 10.3389/fchem.2025.1553975

**Published:** 2025-04-01

**Authors:** Francisco Delgado, Andrés Benítez, Lourdes Gotopo, Angel H. Romero

**Affiliations:** ^1^ Grupo de Química Orgánica Medicinal, Facultad de Ciencias, Universidad de la República, Montevideo, Uruguay; ^2^ Laboratorio de Biología Redox de Tripanosomatidos, Institut Pasteur de Montevideo, Montevideo, Uruguay; ^3^ Laboratorio de Síntesis Orgánica, Escuela de Química, Facultad de Ciencias, Universidad Central de Venezuela, Caracas, Venezuela

**Keywords:** 4-aminoquinoline, 4,7-dichloroquinoline, S_N_Ar, annulation, Ulmann activation, oxidative dehydrogenation

## Abstract

4-Aminoquinoline is an important scaffold due to its variety of applications in medicinal, synthetic organic, and industrial chemistry. It has gained great relevance for the development of selective and potent leishmanicidal agents targeting parasite mitochondria, agonists and antagonists of Toll-like receptors (TLRs), antimalarials, and anticancer agents. As a consequence of the importance of 4-aminoquinoline as leishmanicidal, the present mini-review article aims to give comprehensive information about the different synthetic alternatives for the synthesis of 4-aminoquinolines, including (i) reactions based on nucleophilic aromatic substitution via conventional heating, microwave, and ultrasound; (ii) one-pot metal-free or metal-catalyzed reactions of inter- and intramolecular cyclization/annulation; (iii) miscellaneous reactions including the dehydrogenative amination of dihydroquinolin-4(*1H*)one and amination via Hartwig–Buchwald cross-coupling or rearrangement reactions.

## 1 Introduction

Quinoline is one of the most important *N*-heteroarenes based on its diverse applications in chemical, medicinal, biological, and industrial fields (Ajani et al., 2022). In particular, the 4-aminoquinolines represent one of the most important quinolinic scaffolds in medicinal chemistry because they are involved in a broad range of biological activities: antimalarials (Sunny et al., 2014; Romero et al., 2015; Valverde et al., 2017), anticancer (Ravindar et al., 2024; Romero et al., 2019a), antileishmanial (Romero and Delgado, 2025; Romero et al., 2019b), antifungal (Senerovic et al., 2020), antiviral (Roldan et al., 2020), antibacterial (Ravindar et al., 2024), anti-inflammatory (Allen and Tiwari, 2021), antianalgesic (Shinkai et al., 2000), anti-Alzheimer (Abdallah, 2024), and antitubercular and as agonists/antagonists of Toll-like receptors (TLRs) (Talukdar et al., 2021). There are many 4-aminoquinoline-based drugs on the market, including chloroquine, hydroxychloroquine, piperaquine, amopyroquine, and amodiaquine as antimalarials (Sunny et al., 2014); neratinib and pelitinib as anticancer agents; bosutinib as oral Src/Abl tyrosine kinase inhibitor; dovitinib as antitumor; amsacrine as antineoplastic (Ravindar et al., 2024); aminacrine as antiseptic; antrafenine, glafenine, and floctafenine as anti-inflammatories (Allen and Tiwari, 2021) and antianalgesics; tacrine as anti-Alzheimer (Abdallah, 2024). Recently, chloroquine (CQ) and hydroxychloroquine (HCQ) have been used for COVID-19 treatment, although more studies are required to understand their real effectiveness (Roldan et al., 2020). In addition, recent studies have shown the potential of the 4-aminoquinoline for the design of agonist and/or antagonist of TLRs (Talukdar et al., 2021) (Supplementary Chart S1).

4-Aminoquinoline has shown a high versatility for accumulating into the lysosome, vacuoles, and mitochondria, and other compartments like acidocalcisomes, which can be key for the design of specific types of chemotherapeutic agents like leishmanicidals (Romero and Delgado, 2025), anticancer (Bartel et al., 2019), and antimalarials (Biagini et al., 2003). The presence of two basic moieties, a weak base concerning the quinolinic nitrogen (*pK*_*a*_ ∼ 6) and a tertiary alkylamine placed at the 4-substituted alkyl/aryl chain (*pK*_*a*_ ∼ 7–8), are essential for accumulation into macrophage lysosome as well as into the mitochondria of the parasite as the protonated form after protonation under an acidic lysosome pH. Meanwhile, the presence of lipophilic chains is crucially important for passing through the macrophage lysosome and parasite mitochondria membranes. Thus, the presence of 4-aminoalkyl/aryl chains as well as hydrocarbon chains is key to promoting a selective leishmanicidal response either in infected *in vitro* or *in vivo* models.

Herein, we provide a comprehensive mini-review of the main synthetic strategies to access the 4-aminoquinolines, boarding from classical (Weyesa and Mulugeta, 2020) to modern strategies, which could be of great relevance for the development of leishmanicidal agents. Then, the present review is divided into three sections: (i) reactions based on nucleophilic aromatic substitution via conventional heating, microwave, and ultrasound; (ii) one-pot metal-free or metal-catalyzed reactions of inter- and intramolecular cyclization/annulation; and (iii) miscellaneous reactions.

## 2 Synthetic strategies

### 2.1 Strategies based on nucleophilic aromatic substitution

**SCHEME 1:**
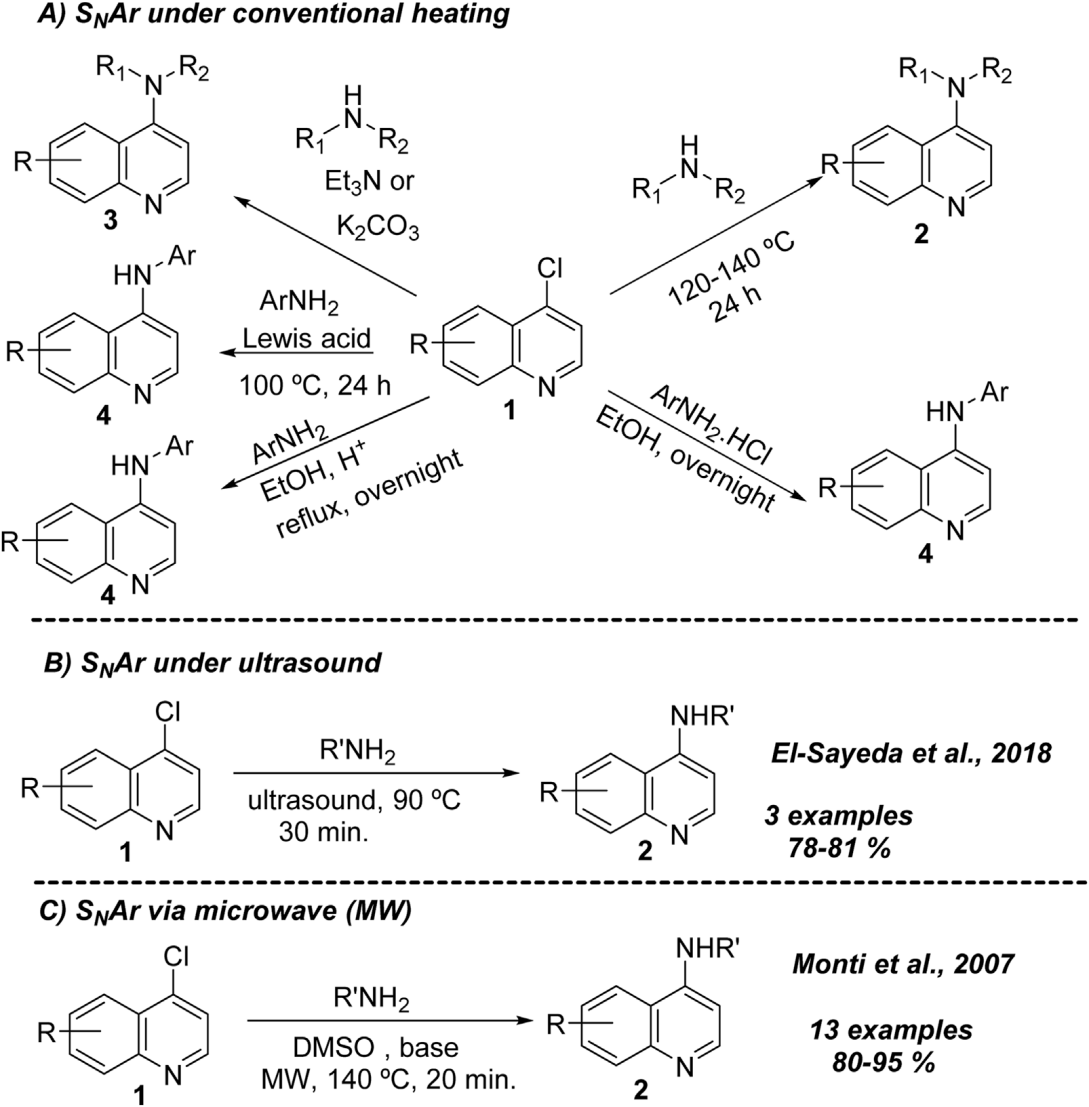
General typical strategies for the preparation of 4-aminoquinolines via S_N_Ar reactions under conventional **(A)** and novel strategies based on ultrasound **(B)** or microwave **(C)**.

Most known 4-aminoquinolines are prepared from the direct coupling between 4-chloroquinolines and an amine-substrate. This type of reaction operates under a nucleophilic aromatic substitution (S_N_Ar), where the carbon placed at the 4-position is attacked by the nucleophile, and then the chlorine is replaced as a leaving group. Under conventional heating, there are at least five different protocols to access 4-aminoquinoline from 4-chloroquinolines (Scheme 1A). The first consists of the direct coupling between the 4-chloroquinoline **1** and alkylamine in alcohol or DMF under extreme conditions (T > 120°C, t > 24 h) (Antinarelli et al., 2015; Konstantinović et al., 2018). The strategy is more suitable for alkylamines than for anilines, being better for secondary dialkylamine than primary alkylamines. This type of protocol can also be applicable in the absence of solvent using an excess of alkylamine as solvent at high temperatures and prolonged time (Yousuf et al., 2015). A second strategy consists of the use of a base like triethylamine or carbonate/bicarbonate, alone or in combination, which has allowed improvement of the reaction yield and reactivity of the 4-chloroquinoline **1** toward a broader group of alkylamines (Coimbra et al., 2016). However, poor yields are found by using anilines.

A third strategy consisted of the use of a catalytic amount of a Brønsted acid (e.g., hydrochloridric acid) or a Lewis acid, which has allowed the incorporation of aniline at the 4-position with high reaction yields and an easy operational procedure (Romero et al., 2015; Valverde et al., 2017). However, the procedure is not convenient for alkylamines because of its possible protonation with acid. The anilines can also be incorporated in 4-chloroquinolines using a Lewis acid (fourth strategy; Cabrera and Márquez, 1999). A fifth strategy consists of using aniline hydrochlorhydrate against 4-chloroquinolines, giving a good yield of approximately 70%–80% (Guglielmo et al., 2009).

Beyond conventional heating, the use of ultrasound and microwave has allowed access to 4-aminoquinolines. Under ultrasound, a series of new 4-aminoquinolines were prepared using a small group of nucleophiles of benzene-1,2-diamines, semicarbazides, and amino-*N*-heteroarenes (3 examples) against 4,7-dichloroquinolines, giving the corresponding products in good to excellent reaction yields of 78%–81% (Aboelnaga and EL-Sayeda, 2018) (Scheme 1B). Under microwave, 4-aminoquinolines can be prepared using 4,7-dichloroquinoline and a variety of alkylamines (primary and secondary), anilines, and amine-*N*-heteroarenes, giving the desired products in good yields (80%–95%) (Melato et al., 2007) (Scheme 1C). The reaction operated in DMSO better than ethanol and acetonitrile at 140°C or 180°C in short times (20–30 min). A base auxiliary was needed when secondary amines were used as nucleophiles, whereas a stronger base, like sodium hydroxide, was required when an aryl/heteroarylamine was used. No extra base was needed when a primary amine was used as a nucleophile.

### 2.2 Strategies based on one-pot inter- and intramolecular cyclization

This type of strategy seeks the preparation of 4-aminoquinolines from inter- and intramolecular cyclization/annulation of any type of substituted aniline (e.g., 2-aminobenzonitriles, 2-formylaniline, ynamines, etc.) with acrylate type compounds (e.g., alkyl/arylisocyanides) and a substituted acetylene (Supplementary Schemes S2–S18). These strategies allow the introduction of different types of functionalization at the 2-, 3-, or benzo-position in a 4-aminoquinoline core, which is a remarkable advantage over the typical strategy based on S_N_Ar because a few 4-chloroquinolines are available in the market as starting material.

In 1992, a palladium-catalyzed multicomponent domino reaction for the synthesis of 2-aryl-4-dialkylaminoquinolines **8** was reported. The reaction allowed obtaining 4-aminoquinolines in moderate to good yields through tandem conjugate addition/cyclization reactions of the *in situ* generated *β*-(2 aminoaryl/heteroaryl)-*α,β*-ynones **7** with amines from ethynylarylamine **5** passing by **6** (Supplementary Scheme S2) (Torii et al., 1992). The reaction needed the use of ethynylarylamines, aryliodides, carbon monoxide (18 bar), and dialkylamines or alkylamines, triethylamine as a base, and a PdCl_2_(PPh_3_)_2_ catalyst in THF at 70° C for 24 h. The procedure was effective only with the use of secondary amines.

In 2005, a modified Torii´s palladium catalyst for the preparation of substituted 2-aryl-4-amino-quinolines **11** was developed (Abbiati et al., 2005). Similarly, the scope of the reaction was examined using carbon monoxide, two 2-ethynyl-arylamines **9**, four aryliodides **10**, and 10 primary amines as substrates (Supplementary Scheme S3). The reaction ran efficiently with the use of Pd(OAc)_2_ in combination with a tri (*o*-tolyl)phosphine (TPP) ligand. A discrete decrease in the yield was found for sole Pd(OAc)_2_ or PdCl_2_/TPP catalyst, whereas a higher decrease was observed with the use of bidentate phosphine ligands. The reaction was compatible with a variety of alkyl/aryl amines and was more efficient for alkylamines. No substantial differences were noted as a function of the 2-ethynyl-arylamine and aryliodide.

In 2012, 2-halocarbon-3-phosphonyl-4-aminoquinolines **14** were synthesized from the coupling between 2-aminobenzonitriles **12** and halocarbon-alkynylphosphonates **13** through an intermolecular annulation (Supplementary Scheme S4) (Duda et al., 2012). The reaction proceeded efficiently with the use of K_2_CO_3_ in toluene at 112°C for 12 h. The use of the base was pivotal, with no reaction in the absence of the base. A decrease was found by using organic bases such as triethylamine, DBU, or DABCO. Toluene displayed better reaction yields than dichloromethane, benzene, or DMF. The reaction was compatible with a variety of substituents into 2-aminobenzonitrile and halocarbon substituents into acetylene, giving good yields (62%–95%) in most of the studied cases.

In 2013, 2-aryl-4-terbutylaminoquinolines **16** were prepared from the intermolecular cyclization between *N*-aryl-ethan-1-imine **15** and terbutyl-isocyanide (Supplementary Scheme S5) (Vlaar et al., 2013). The best conditions consisted of Pd(OAc)_2_ in combination with pivalic acid in the presence of molecular sieves in toluene at 100°C under an oxygen atmosphere, although the conversion to product was discretely low in most cases (yields between 19% and 27%). No improvements were found by using other oxidants like CuCl_2_, Cu(OAc)_2_, AgOAc, benzoquinone, or K_2_S_2_O_8_, solvents (THF, DMSO, DMF, MeCN, dioxane, DCE, or DME), palladium catalysts [PdCl_2_, Pd(CF_3_COO)_2_, Pd (MeCN)_2_Cl_2_], or any coordinating additives, such as 1,10-phenanthroline and pyridine.

In the same year, a copper(I) catalyzed protocol using alkynyl-aryl-imines **17** and sulfonylazides **18** disclosed 2-aryl-4-sulfoaminoquinoline **19** (Supplementary Scheme S6) (Cheng and Cui, 2013). The optimal conditions consist of the use of CuI as a catalyst and K_2_CO_3_ in dichloromethane at room temperature for 12 h. Dichloromethane displayed better reaction yields than other solvents like chloroform, THF, dioxane, acetonitrile, and DMF, whereas K_2_CO_3_ (1.5 eq.) displayed better yields than other bases like triethylamine, pyridine, Cs_2_CO_3_, KHCO_3_, or Na_2_CO_3_. The reaction was compatible with a variety of substituents into 2-aminobenzonitrile and halocarbon substituents into acetylene, giving good yields (56%–82%) in most studied cases. No compatibility was found for an alkynyl-bearing *o*-substituted aryl moiety.

In the next year, palladium (II) catalyzed a cascade reaction between 2-iodide-aryl-enaminones **20** and alkylisocyanide and allowed the synthesis of a series of 2-carbonyl-4-aminoquinolines **21** (Supplementary Scheme S7) (Gu et al., 2014). The coupling proceeded efficiently with the use of Pd (dppf)_2_Cl_2_ and Cs_2_CO_3_ in dioxane at 110°C for 12 h. Pd (dppf)_2_Cl_2_ was identified as the best catalyst over Pd(PPh_3_)_2_Cl_2_, PdCl_2_, Pd (dba)_2_, Pd(OAc)_2_ or Pd(PPh_3_)_2_. The selection of the base was crucially important, finding the lowest yields by using triethylamine, NaOAc, KOAc, Na_2_CO_3_, phosphates, or DABCO. The reaction was highly efficient when cyclic ketone substrates were used, giving reaction yields higher than 80% for most of the studied cases. The reaction is compatible with a variety of substituents into the aryl ring of aniline substrate. Terbutyl-isocyanide was significantly more compatible than other alkyl-isocyanides. No reaction was detected when using benzylisocyanide or phenylisocyanide.

Then, in 2015, an extended strategy for the preparation of 3-carbonyl-4-aminoquinolines **23** through a palladium-catalyzed intermolecular oxidative cyclization of *N*-arylenamines **22** with isocyanides via double *sp* ^*2*^ C-H bonds cleavage was reported (Supplementary Scheme S8) (Zheng et al., 2015). The optimal conditions involved the use of Pd(OAc)_2_, 1,10-phenanthroline, Cs_2_CO_3_ as the base, and Cu(OAc)_2_ as an oxidant in DCE at 80°C for 16 h. The reaction was compatible with a broad aryl scope for *N*-arylenamine, expecting those bearing *o*-substitution that provided low yields (25%–30%). The reaction was only compatible with alkyl substitution into isocyanide substrate because aryl substitution provided traces of product or no reaction. Adding a terbutyl chain into isocyanides was more compatible than adding cyclohexyl or adamantyl.

In the same year, an azahetero-Diels–Alder reaction was developed for preparing dimethyl 2,3-dicarboxylate-4-aminoquinolines **26** from 2*H*-indazole **25** and dimethylacetalenedicarboxylate (Supplementary Scheme S9) (Vidyacharan et al., 2015). First, the 2*H*-indazoles were prepared from the coupling between 2-azidobenzaldehyde **24** with the nucleophile (*e.g.*, alkylamines, anilines, and benzylamines) at 120°C for 1.5–3 h. With the 2*H*-indazole, the best reaction conditions for the coupling with the dimethylacetalenedicarboxylate operated in benzene at 80°C for 30 h. A significant decrease in the reaction yield was appreciated in polar and protic solvents such as DMSO, methanol, or water. From the synthesized 2*H*-indazoles, a variety of substituted dimethyl 2,3-dicarboxylate 4-aminoquinolines were produced in good to excellent yields (55%–75%). The procedure allows the introduction of *N*-benzylamines, anilines, and alkylamines at the 4-position of the quinoline ring.

Later, in 2016, a [2 + 2] annulation strategy was developed for the synthesis of 2,3-disubstituted-4-aminoquinolines **29** from substituted carboxanilide **28** and sulfonyl ynamides **27** (Supplementary Scheme S10) (Wezeman et al., 2016). The reaction was operationally simple and compatible with a variety of substituents R_1_, R_2_, and R_3_ in sulfonyl ynamides and R_4_ and R_5_ substitutions in carbozanilide. It was promoted by triflic acid in the presence of 2-chloropyridine in dichloromethane at −78°C in the first stage to room temperature for 1 h.

In 2017, a multicomponent reaction between phenylacetylenes **30**, alkyl/aryl-sulfonylazides **31**, and 2-aminobenzonitrile **32** was reported to access 2-aminosulfonyl-3-substituted-4-aminoquinolines **33** (Supplementary Scheme S11) (Yi et al., 2017). The reaction showed a good scope tolerance, giving the desired product in moderate to excellent reaction yields (65%–74%) for most cases. The reaction conditions required the use of triethylamine and catalytic CuI in dichloromethane at 80 C for 4 h under a nitrogen atmosphere. The procedure allows the introduction of an aryl and a sulfoamino group at the 3- and 2-position, respectively, of the quinoline ring.

Alternatively, using 2-aminobenzonitriles **34** in combination with 1,1,1-trichloro-4-ethoxybut-3-enone **35** has allowed accessing ethyl 2-carboxylate-4-aminoquinolines **37** through a three-step route (Supplementary Scheme S12) (Lavrard et al., 2017). The first reaction consists of a condensation to afford the corresponding enaminones **36** (70%–97%), which can easily cycle to give the corresponding 4-aminoquinoline under acid conditions using trifluoromethanesulfonic acid. Then, in the presence of sodium ethoxide in ethanol, the trichloromethyl moiety is replaced by ethoxy group to give the desired ethyl 2-carboxylate-4-aminoquinolines in good to excellent yields (60%–89%) for most of the cases with good group tolerance.

Following, in 2017, a copper (II)-catalyzed reaction, based on aerobic oxidative desulfitative 6π electrocyclization in *N*-aryl-imino ketene *N,S*-acetal as substrates **39,** was developed to access 2-methyl-3-carboxylate-4-anilinoquinolines **40** (Supplementary Scheme S13) (Shi et al., 2017). Substrate **39** was prepared from the reaction between the readily available ketene *S,S*-acetals **38** with anilines at 80°C in toluene in the presence of triflic acid. With *N*-aryl-imino ketene *N,S*-acetals **39** in hand, the reaction proceeded using CuCl_2_, 1,10-phenanthroline as a ligand and K_2_CO_3_ in toluene at 80°C for 12 h, allowing the desired 4-anilinoquinolines in good yields with good group tolerance. Similar yields were found by using CuI, CuO, and CuBr_2_. Lower yields were found in chloroform, ethanol, DMF, or dioxane.

In the same year, a multicomponent reaction via a copper-catalyzed [2 + 2 + 2] annulation was reported for the synthesis of 2,3-disubstituted 4-aminoquinolines **44** from substituted benzonitriles **41**, aryl-mesyliodinium salt **42** and ynamides **43** (Supplementary Scheme S14) (Oh et al., 2017). The strategy was operationally simple, with a high atom economy and compatible with a variety of substrates for benzonitrile and aryliodonium salt. For the ynamide, only *N*-benzyl-substitution provided the best reaction yields compared to *N*-methyl substitution. The reaction was catalyzed by CuTC over other species of copper (I), such as CuPF_6_, Cu(OAc), or CuCl. The optimal conditions consist of CuTC, ethylacetate as a solvent, and molecular sieves at 75°C for 3 h.

In 2018, 2-(alkyl/aryl)-4-aminoquinolines **48** were synthesized through a three-component reaction via an imidoylative Sonogashira/cyclization cascade (Supplementary Scheme S15) (Collet et al., 2018). The reaction operates through a carbon-halogen activation using Pd(OAc)_2_, Xantphos as the ligand, CuBr as a catalyst auxiliary, and Cs_2_CO_3_ in DMF at 90°C for 16 h and then, HCl addition under stirring at room temperature for 15 min. DMF was recognized as the best solvent among DMSO, dioxane, and toluene. The reaction was highly compatible with a variety of aryl/alkyl-acetylenes **45**, alkyl-isocyanides **46**, and 2-bromoaniline **47**. Aryl isocyanides were not compatible with the procedure. The procedure is convenient for the introduction of an alkyl diamine chain at the 4-position of the quinoline ring.

Later, in 2018, a gold-catalyzed *syn*-1,2-difunctionalization of ynamides via nitrile activation was introduced for the synthesis of 2-aminotosyl-3-aryl-4-aminoquinolines **51** from the coupling between 2-aminobenzonitriles **49** and aryl-acetylenes **50** (Supplementary Scheme S16) (Vanjari et al., 2018). The best reaction conditions consisted of the use of a Ph_3_PAuNTf_2_ catalyst in dioxane at 80 °C for 7 h. Dioxane showed better reaction yields than dichloroethane or acetone, whereas moderate yields were noted in JohnPhosAu (MeCN)SbF_6_, CyJohnPhosAuNTf_2_, and IprAuNTf_2_. No reaction or low yields (˂10%) were found using a gold catalyst, such as Cu(OAc)_2_, Pd(OAc)_2_, and AgNTf_2_. The reaction was compatible with a variety of aryl substituents with good yields (72%–93%) for aryl-acetylenes, whereas acetylenes bearing alkyl chains displayed the lowest yields (26%–40%), including no reaction for acetylenes bearing thiophene moiety.

In 2021, novel 2-thiomethyl-3-cyane-4-aminoquinolines **53** were prepared from the *N*-heteroannulation of *β*-anilino-*β*-(methylthio)acrylonitriles **52** using triflic acid (Supplementary Scheme S17) (Bandyopadhyay et al., 2021). The latter substrates were prepared from the reaction between aryl cyanides with aryl-isothiocyanates in DMF in the presence of NaH and methyl iodide from 0°C during the first stage of the reaction until room temperature. The reaction allows the desired substrate to be obtained with good scope tolerance and good reaction yields (50%–89%). With *β*-anilino-*β*-(methylthio)acrylonitriles in hand, the reaction condition implied the use of triflic acid as a key additive for facilitating the *N*-heteroannulation under 60°C for 4 h and 60 h, allowing obtaining 2-thiomethyl-3-cyane-4-aminoquinolines in good yields with good group tolerance.

In the same year, a novel aza-Michael addition/intramolecular annulation was implemented for the synthesis of polysubstituted 4-aminoquinolines **56** from ynones **54** and 2-aminobenzonitriles **55** (Supplementary Scheme S18) (Kumar et al., 2021). The reaction allowed access to a variety of 2-substituted-3-carbonyl-4-aminoquinolines in good to excellent yields. The strategy was operationally simple and scalable, had a high atom economy, and was compatible with a variety of substrates and different substituents R_1_, R_2_, and R_3_ in the acetylenes and 2-aminobenzonitrile substrates. The reaction occurred by using potassium terbutoxide under 100°C in DMSO for 1 h. Lower reaction yields were obtained in DMF, NMP, and dioxane, and no reaction occurred in toluene and ethanol. KO*t*-Bu displayed better reaction yields than LiO*t*-Bu, Cs_2_CO_3_, KOH, NaOH, and K_3_PO_4_.

### 2.3 Miscellaneous

In this section, some varied strategies beyond inter- or intramolecular cyclization/annulation or S_N_Ar reactions are shown, which imply intermolecular rearrangements, direct amination via C-halogen activation, and other ways of S_N_Ar or oxidative amination.

**FIGURE 1:**
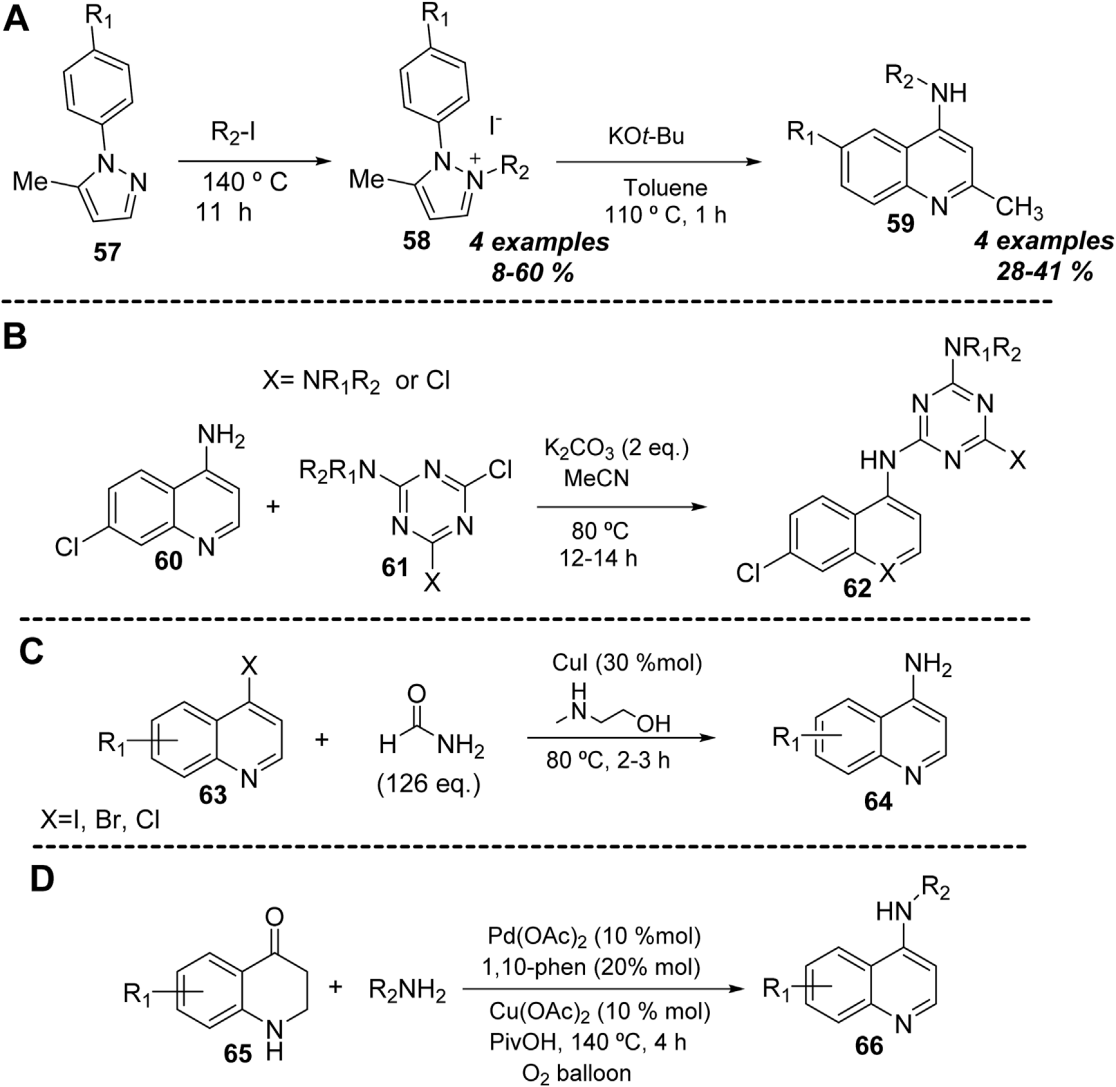
Miscellaneous reactions for the synthesis of 4-aminoquinolines **(A–D)**.

In 2010, a novel procedure for the preparation of 4-aminoquinolines **59** from 1-phenyl-substituted pyrazolium salts **58** was reported. The reaction proceeds through a rearrangement through sequential deprotonation of pyrazol-3-ylidenes, ring-opening, ring-closure, and final tautomerization to disclose the substituted 4-aminoquinolines (Figure 1A) (Dreger et al., 2010). The reaction proceeded using a strong base (KO*t*-Bu) in toluene under heating for 1 h. The protocol allowed obtaining a series of 4-aminoquinolines in low yields (28%–41%). Only higher yields were found for 1-naphtlypyrazolium to give benzo [*h*]quinolin-4-amine. The 1-phenyl-substituted pyrazolium salts were prepared from *N*-alkylation of 1-phenyl-pyrazoles **57** using alkyliodides.

From the classical strategies, it is possible to access 4-(substituted amino)quinoline **62** using 4-aminoquinoline **60** (Manohar et al., 2010). It is probable that this type of strategy would only be compatible with stronger electrophilic sources (e.g., chloro-1,3,5-triazines **61**). The reaction proceeds with the use of K_2_CO_3_ in acetonitrile under reflux for 12–14 h, giving good reaction yields (49%–65%) (Figure 1B).

An example of Hartwig–Buchwald coupling via carbon-halide activation was reported in 2014 (Aillerie et al., 2014). The protocol allows the introduction of an unsubstituted amine at the 4-position of quinoline to give 4-aminoquinolines **64** from 4-halidequinolines **63**. The reaction proceeds through a carbon-halide activation using CuI and formamide as the amine source, which is released *in situ* in the presence of 2-aminoethanol. The reaction was compatible with a broad scope concerning the quinoline core. The reaction showed the best yields for iodide substrates over a bromide substrate, whereas the chloride substrate did not react (Figure 1C).

Recently, in 2023, a palladium-catalyzed dehydrogenative aromatization was implemented for the synthesis of 4-aminoquinolines **66** from 2,3-dihydroquinolin-4(1*H*)-one **65** with amines (Chen et al., 2023). The reaction proceeded by using Pd(OAc)_2_ in the presence of Cu(OAc)_2_ as the oxidant, 1,10-phenanthroline as the ligand, and pivalic acid as the solvent at 140°C for 4 h using an oxygen balloon. The reaction showed an excellent group tolerance, either for alkyl/aryl amines or for substitution at the benzo-quinoline core, which allowed obtaining known antimalarial drugs, such as chloroquine and amodiaquine, in good yields (Figure 1D).

In summary, the present mini-review showed diverse alternatives for the preparation of 4-aminoquinolines, which are based on conventional strategies of S_N_Ar that are the most practical but present some disadvantages concerning the availability of quinoline substrates. As an alternative, intermolecular or intramolecular cyclization/annulation using 2-aminobenzonitrile or analogs allowed accessing a variety of 4-aminoquinolines, although the synthesized compounds are characterized by bearing a 2- or 3-substitution. Then, the dehydrogenative aromatization and subsequent amination using any type of alkylamine or anilines, catalyzed by Pd (II) or Cu (II), emerged as a suitable alternative, which allowed the preparation of popular 4-aminoquinolines such as chloroquine and amodiaquine in good yields.
