# Supplementary material for: 4-Aminoquinoline: a comprehensive review of synthetic strategies
Source: Front Chem. 2025 Apr 1;13:1553975. doi: 10.3389/fchem.2025.1553975 (PMC12023255; doi:10.3389/fchem.2025.1553975)
Supplement: Supplementary file 1 [file DataSheet1.docx]

**SUPPORTING INFORMATION MATERIAL:**

**4-Aminoquinoline: A Comprehensive Review about Synthetic Strategies**

Francisco Delgado,^1,2^ Andrés Benítez,^1^ Lourdes Gotopo,^3^ Angel H. Romero^1*^

*^1^Grupo de Química Orgánica Medicinal, Facultad de Ciencias, Universidad de la República, 11400 Montevideo, Uruguay.*

*^2^Laboratorio de Biología Redox de Tripanosomatidos. Institut Pasteur de Montevideo. Montevideo, Uruguay.*

*^3^Laboratorio de Síntesis Orgánica, Escuela de Química, Facultad de Ciencias, Universidad Central de Venezuela, Los Chaguaramos, Caracas, Venezuela.*

*** Correspondence:**Angel H. Romero (E-mail: [angel.ucv.usb@gmail.com](mailto:angel.ucv.usb@gmail.com); [aromero@fcien.edu.uy](mailto:aromero@fcien.edu.uy))

**Chart 1.** Some relevant chemotherapeutic drugs/agents based on 4-aminoquinolines: antimalarials; leishmanicidal drugs/agents; anticancer drugs/agents and TLR agonist/antagonists.

**Scheme 2**

**Scheme 3**

**Scheme 4**

**Scheme 5**

**Scheme 6**

**Scheme 7**

**Scheme 8**

**Scheme 9**

**Scheme 10**

**Scheme 11**

**Scheme 12**

**Scheme 13**

**Scheme 14**

**Scheme 15**

**Scheme 16**

**Scheme 17**

**Scheme 18**
